# Influence of Chronic Obstructive Pulmonary Disease and Moderate-To-Severe Sleep Apnoea in Overnight Cardiac Autonomic Modulation: Time, Frequency and Non-Linear Analyses

**DOI:** 10.3390/e21040381

**Published:** 2019-04-09

**Authors:** Daniel Álvarez, Ana Sánchez-Fernández, Ana M. Andrés-Blanco, Gonzalo C. Gutiérrez-Tobal, Fernando Vaquerizo-Villar, Verónica Barroso-García, Roberto Hornero, Félix del Campo

**Affiliations:** 1 Sleep-Ventilation Unit, Pneumology Service, Río Hortega University Hospital, c/ Dulzaina 2, 47012 Valladolid, Spain; 2 Biomedical Engineering Group, University of Valladolid, Paseo de Belén 15, 47011 Valladolid, Spain

**Keywords:** chronic obstructive pulmonary disease, obstructive sleep apnoea syndrome, pulse rate variability, non-linear analysis, sample entropy

## Abstract

Chronic obstructive pulmonary disease (COPD) is one of the most prevalent lung diseases worldwide. COPD patients show major dysfunction in cardiac autonomic modulation due to sustained hypoxaemia, which has been significantly related to higher risk of cardiovascular disease. Obstructive sleep apnoea syndrome (OSAS) is a frequent comorbidity in COPD patients. It has been found that patients suffering from both COPD and OSAS simultaneously, the so-called overlap syndrome, have notably higher morbidity and mortality. Heart rate variability (HRV) has demonstrated to be useful to assess changes in autonomic functioning in different clinical conditions. However, there is still little scientific evidence on the magnitude of changes in cardiovascular dynamics elicited by the combined effect of both respiratory diseases, particularly during sleep, when apnoeic events occur. In this regard, we hypothesised that a non-linear analysis is able to provide further insight into long-term dynamics of overnight cardiovascular modulation. Accordingly, this study is aimed at assessing the usefulness of sample entropy (SampEn) to distinguish changes in overnight pulse rate variability (PRV) recordings among three patient groups while sleeping: COPD, moderate-to-severe OSAS, and overlap syndrome. In order to achieve this goal, a population composed of 297 patients were studied: 22 with COPD alone, 213 showing moderate-to-severe OSAS, and 62 with COPD and moderate-to-severe OSAS simultaneously (COPD+OSAS). Cardiovascular dynamics were analysed using pulse rate (PR) recordings from unattended pulse oximetry carried out at patients’ home. Conventional time- and frequency- domain analyses were performed to characterise sympathetic and parasympathetic activation of the nervous system, while SampEn was applied to quantify long-term changes in irregularity. Our analyses revealed that overnight PRV recordings from COPD+OSAS patients were significantly more irregular (higher SampEn) than those from patients with COPD alone (0.267 [0.210–0.407] vs. 0.212 [0.151–0.267]; *p* < 0.05) due to recurrent apnoeic events during the night. Similarly, COPD + OSAS patients also showed significantly higher irregularity in PRV during the night than subjects with OSAS alone (0.267 [0.210–0.407] vs. 0.241 [0.189–0.325]; *p* = 0.05), which suggests that the cumulative effect of both diseases increases disorganization of pulse rate while sleeping. On the other hand, no statistical significant differences were found between COPD and COPD + OSAS patients when traditional frequency bands (LF and HF) were analysed. We conclude that SampEn is able to properly quantify changes in overnight cardiovascular dynamics of patients with overlap syndrome, which could be useful to assess cardiovascular impairment in COPD patients due to the presence of concomitant OSAS.

## 1. Introduction

Chronic obstructive pulmonary disease (COPD) is characterised by a persistent and commonly progressive airflow limitation consequent to an abnormal inflammatory response of the airway and lung tissue due to noxious particles and gases [1]. According to a recent meta-analysis [2], COPD is among the most common lung diseases, with an estimated prevalence of 9%, becoming a major health problem around the world. Despite being a preventable and treatable condition, COPD is currently among the leading causes of mortality and morbidity worldwide. COPD is ranked eighth among the conditions causing disability globally [3] and is the third leading cause of death among adults older than 40 years old [4].

A substantial number of patients suffering from COPD have major comorbidities, which is linked with increased mortality [5]. In this regard, sleep disorders are frequently present in COPD patients, particularly obstructive sleep apnoea syndrome (OSAS). Individually, both diseases are linked with a wide range of physiological disturbances, such as hypoxemia and inflammation [6]. Nevertheless, COPD and OSAS do not share the same mechanisms leading to inadequate gas exchange. While COPD is characterised by a chronic baseline hypoxaemia due to a permanent airflow limitation, OSAS patients show recurrent desaturations that lead to a pattern of intermittent hypoxaemia during the night [7]. Therefore, the pattern of intermittent desaturation superposes to a pathological baseline hypoxaemia in patients showing both diseases.

The coexistence of COPD and OSAS, the so-called overlap syndrome, leads to major social and healthcare-related consequences, mostly in the context of cardiovascular disease [8]. In this regard, heart rate variability (HRV) analysis, which is commonly used to assess autonomic imbalances linked with diseased states, has been found to provide relevant information on the effect of overlap syndrome on cardiac regulation [9,10]. Patients simultaneously showing both conditions have significant imbalances in cardiac autonomic modulation compared to those with COPD or OSA alone, such as higher sympathetic and lower parasympathetic activity [7]. COPD patients show reduced daytime HRV when compared to healthy subjects [10,11,12] and a recent study evidenced even lower resting HRV in patients with overlap syndrome, which is related to increased risk of cardiac disease [9]. Accordingly, the revised Global Initiative for Chronic Obstructive Lung Disease (GOLD) and the American Thoracic Society (ATS) highlight the need for controlling the impact of comorbid conditions in COPD patients, especially sleep-disordered breathing [1,13]. However, further research is still needed on the combined effect of both respiratory diseases on HRV dynamics, particularly during sleep, when apnoeic events arise.

Abnormal HRV dynamics are associated with several pathological conditions [14]. The assessment of HRV is commonly accomplished using traditional time and frequency domain measures, such as standard deviation of RR intervals (SDNN), the root mean square of successive differences (RMSSD) and the spectral power in the low (LF: 0.04–0.15 Hz) and high (HF: 0.15–0.40 Hz) frequency bands of the RR time series [15]. However, conventional methods are affected by nonlinearities and non-stationarities commonly present in biological systems, preventing long-term analyses from being properly performed. On the other hand, non-linear methods have been found to overcome these drawbacks, providing essential and complementary information on physiological time series dynamics. Among non-linear methods, entropy is probably the most widely used metric to assess irregularity of physiological signals. Particularly, sample entropy (SampEn) has been found to provide further insight into several major diseases and physiological conditions, such as Alzheimer’s disease [16], diabetes [17], atrial fibrillation [18], and aging [19]. Moreover, SampEn has been previously used to analyse cardiorespiratory-related signals in the context of OSAS [20,21,22] and COPD [12,23,24,25]. Nevertheless, few studies have used non-linear measures to assess changes in HRV due to the simultaneous presence of COPD and OSAS [9,26]. In the present study, we hypothesized that SampEn could provide additional and relevant information on the changes in overnight HRV dynamics of COPD patients due to the presence of concomitant moderate-to-severe OSAS. Accordingly, our aim was to assess differences in irregularity of overnight pulse rate variability (PRV) from nocturnal pulse oximetry of COPD patients with and without moderate-to-severe OSAS.

## 2. Material and Methods

### 2.1. Population under Study

A dataset composed of 297 patients showing moderate-to-high clinical suspicion of suffering from sleep apnoea were analysed: 22 with COPD alone (COPD group), 213 showing moderate-to-severe OSAS (OSAS group), and 62 with COPD and concomitant moderate-to-severe OSAS (COPD + OSAS group). Individuals with a confirmed diagnosis of COPD who visited the pneumology outpatient facilities and showed symptoms of suffering from sleep disturbance breathing were asked to participate in the study. The recommendations of the GOLD task force were used for COPD diagnosis [1]. Accordingly, pre- and post-bronchodilator spirometry, lung volumes, and lung diffusion capacity were conducted for complete pulmonary function evaluation (Master screen PFT, Jaeger). A positive diagnosis of COPD was established for patients with a smoking history (current or ex-smokers) of at least 10 packs/year, respiratory symptoms and showing a post-bronchodilator spirometry with forced expired volume in 1 s to forced vital capacity ratio (FEV_1_/FVC) < 70% [1]. On the other hand, individuals without COPD derived consecutively to the sleep unit due to suspicion of OSAS were also involved in the study. Patients with a previous diagnosis and/or treatment for OSAS or showing additional sleep disorders were excluded from the study. In addition, patients showing need for oxygen therapy due to respiratory failure were also excluded. The research was conducted according to the principles expressed in the Declaration of Helsinki. Accordingly, all patients were informed to participate in the study and signed an informed consent. The Ethics Committee of the Río Hortega University Hospital approved the protocol (approval number: CEIC 7/13).

All subjects were referred for a polysomnography (PSG) study in order to confirm or discard moderate-to-severe OSAS. In-laboratory nocturnal PSG was carried out for standard electroencephalographic and cardiorespiratory assessment using a polysomnograph E-series (Compumedics Limited, Victoria, Australia). A single expert scored each PSG according to the American Academy of Sleep Medicine (AASM) rules in order to derive the apnoea-hypopnoea index (AHI) [27]. All PSGs with a total sleep time (TST) < 3 h were withdrawn from the study (insufficient data to assess sleep) [28]. A cut-off of 15 events/h were used for a positive diagnosis of moderate-to-severe OSAS.

Table 1 shows the socio-demographic and clinical variables of the groups under study. Male gender was predominant in the three groups. Patients with COPD + OSAS were older than patients with OSAS or COPD alone. There were no significant differences between groups in terms of body mass index (BMI). Regarding PSG-derived indexes, patients with OSAS (OSAS alone or overlap) showed significantly higher AHI and number of desaturations equal or greater than 3% from baseline per hour of sleep (ODI3) than COPD patients. The COPD+OSAS group showed significantly higher cumulative time with a saturation under 90% (CT90) than the groups with COPD or OSAS alone. Similarly, COPD + OSAS patients showed significantly lower minimum saturation than patients with COPD or OSAS alone, which agrees with the deeper desaturations typical of overlap patients during the night [7,8]. On the other hand, patients in the OSAS group had significantly higher baseline saturation than patients with COPD or overlap syndrome. Similarly, patients with OSAS alone had lower minimum saturation than COPD patients. Finally, patients with COPD + OSAS showed significantly lower overnight average saturation than patients with COPD or OSAS alone. Regarding the pulmonary function, no significant differences between COPD patients with and without OSAS were found in terms of forced expiratory volume in 1 s (FEV_1_) improvement, forced vital capacity (FVC) improvement, and the ratio FEV_1_/FVC after post-bronchodilator spirometry.

In the COPD group, five patients (22.7%) showed an AHI < 5 events/h and 17 patients (77.3%) showed 5 ≤ AHI < 15 events/h from PSG, which excluded moderate-to-severe OSAS. A total of five patients (22.7%) reported witnessed apnoeas and 13 (59.1%) loud snoring. The average Epworth score was 10.4 points. In the moderate-to-severe OSAS group, 50 patients (23.5%) had 15 ≤ AHI < 30 events/h and 163 (76.5%) AHI ≥ 30 events/h. Within this group, 150 patients (70.4%) reported witnessed apnoeas and 208 (97.7%) loud snoring while sleeping. The average Epworth score was 10.8 points. Finally, 16 patients (25.8%) had 15 ≤ AHI < 30 events/h and 46 (74.2%) AHI ≥ 30 events/h in the overlap group. A total of 25 patients (40.3%) reported witnessed apnoeas and 53 (85.5%) loud snoring, while the average Epworth score was 11.0.

Table 2 summarises the main comorbidities and common therapies of COPD patients. Regarding the presence of diabetes, no statistically significant differences were found among the groups under study. On the other hand, arterial hypertension and ischemic cardiomyopathy were higher in the COPD + OSAS group, which agrees with older as well as potentially more complicated patients. Accordingly, this group also showed a higher use of beta-blockers linked with hypertension and/or cardiovascular disease. No significant differences were found for the use of calcium channel blockers. Regarding common therapies in COPD patients, no statistically significant differences were found between COPD patients with and without OSAS. The use of anticholinergics, β2-agonists, and inhaled corticosteroids, found in the medical record of some OSAS patients, was minimal (significantly minor) and was associated to asthma and/or allergy. Notice that no asthma/allergy-related exacerbation episodes occurred immediately before PSG studies. Finally, it is important to highlight that no patient used positive airway pressure (PAP)-based devices previously to his/her inclusion in the study.

In order to assess cardiovascular dynamics, overnight pulse rate (PR) was recorded at home using a portable pulse oximeter (WristOx2 3150, Nonin Medical, Inc., Plymouth, MI, USA). All the recordings were obtained the day before or the day after in-lab PSG, which was randomly assigned to avoid a potential bias linked with the order of the studies. The PR signal was recorded at a sampling rate of 1 sample per second (1 Hz). Every patient involved in the study received both verbal and written instructions on how to use the pulse oximeter. All patients were asked to go to bed between 10 and 12 PM. PR recordings with a total recording time (TRT) < 3 h due to premature battery depletion, artefacts or voluntary termination by the patient, were discarded [29]. PR time series were automatically scanned to remove zero samples due to patient’s movements and artefacts (abnormal arrhythmia-related pulse-to-pulse intervals). Particularly, all 5-min segments showing more than 1% of samples (pulse-to-pulse interval) outside the range 0.33–1.50 s and differences between consecutive intervals greater than or equal to 0.66 s were excluded from subsequent analyses [30].

### 2.2. PRV Analysis

Three signal processing approaches were applied to analyse overnight PRV: (i) time domain; (ii) frequency domain; and (iii) non-linear analyses. Firstly, common variables in the time domain were computed [15]:

- Average of pulse-to-pulse interval (AVNN). It is a global measure of the inter-beat interval (inverse of pulse rate).
- Standard deviation of pulse-to-pulse interval (SDNN). It provides a global measure of variability.
- Root mean square of successive differences of pulse-to-pulse intervals (RMSSD). It reflects vagal activity.

AVNN, SDNN, and RMSSD are usually computed over the entire recording, providing a long-term measure of the influence of the physiological condition under study, i.e., the influence of combined COPD and OSAS on cardiac dynamics modulation.

Conventional features in the frequency domain were also computed. Firstly, according to Penzel et al. [21,30], every overnight PR recording was resampled at 3.41 Hz using linear interpolation to properly perform frequency domain analyses. Then, the well-known Welch’s method was applied to estimate the power spectral density (PSD) of each PR recording. In order to minimise the problem of non-stationarities, 5-min length epochs were considered [15]. Accordingly, 1024-sample Hamming window with 50% overlap and 2048-point fast Fourier transform (FFT) were used to compute each PSD. Then, the following frequency domain measures were derived [15]:

- Very low frequency power (VLF). It measures rhythms with periodicities between 25 s and 5 min (0.0033–0.04 Hz). VLF reflects vagal and renin-angiotensin system effects on PR. It is usually normalised to the total power of the signal (VLFn).
- Low frequency power (LF). It measures rhythms from 2.5 to 9 cycles per minute (0.04–0.15 Hz). LF is associated with both sympathetic and parasympathetic activity of the nervous system. It is commonly computed as the ratio to the cumulative spectral power in both the LF and HF bands (LFn).
- High frequency power (HF). It captures rhythms with periodicities between 9 and 24 cycles per minute (0.15–0.40 Hz). HF is exclusively modulated by the parasympathetic nervous system reflecting vagal activity. As LF, it is usually measured as the ratio to the spectral power in the LF+HF band (HFn).
- Low frequency to high frequency ratio (LF/HF). It measures the sympathetic component of PRV modulation reflecting the so-called sympathovagal balance.
- Spectral power in the OSAS-related frequency band (OSASF). It reflects changes with periodicities between 30 s and 70 s (0.014–0.033 Hz). The power spectrum in this frequency band has been found to be related to the repetition of apnoeic events during the night [26,28]. It is usually normalised to the total signal power (OSASFn).

Traditionally, cardiorespiratory dynamics are assessed by means of measures in the time and frequency domains. Whilst such conventional approaches account for the magnitude of the changes (amount of variability), non-linear methods are able to measure the underlying organization of physiological signals. In this regard, several works have demonstrated the chaotic behaviour of heart rate recordings [31,32], suggesting the usefulness of non-linear analysis to provide additional information to common time- and frequency-based methods. In this framework, entropy arises as one of the most widely used non-linear measures of physiological signal dynamics. Particularly, sample entropy (SampEn) has been found to be useful in the analysis of cardiorespiratory signals, such as blood oxygen saturation [20,33,34], airflow [35], breathing sounds [36], hear rate variability [22], and pulse rate variability [37].

SampEn was originally proposed by Richman and Moorman to quantify irregularity in time series [38]. It is based on the algorithm of approximate entropy (ApEn), a family of statistics aimed at characterising non-linear dynamics [39]. Despite ApEn has been widely used in several applications, there is a potential bias in the computation of nearby patterns due to self-matching, which could lead to biased irregularity measures [38]. In order to overcome this drawback, SampEn avoids the inclusion of self-matches when quantifying the number of similar patterns in the estimation of probabilities. According to [38], SampEn is computed as follows:

(1) $$SampEn\left(m,r,N\right)=-\ln\frac{A^{m}\left(r\right)}{B^{m}\left(r\right)}$$

where *A^m^* and *B^m^* are the average number of (*m*)-length and (*m*+1)-length segments *X_m_*(*i*) (1 ≤ *i* ≤ *N-m*+1) with *d*[*X_m_*(*i*),*X_m_*(*j*)] ≤ *r* (1 ≤ *j* ≤ *N-m*, *j* ≠ *i*), respectively, and

(2) $$d\left[X_{m}\left(i\right),\;X_{m}\left(j\right)\right]=\underset{k=0,\;\ldots ,m-1}{\max}\left(\left|x\left(i+k\right)-x\left(j+k\right)\right|\right)$$

Briefly, SampEn firstly divides the original data sequence into consecutive segments of length *m*. Then, similarity between each pair of (*m*)-length segments is assessed, considering that a pair of segments are similar if the maximum absolute difference between individual components is less than or equal to a domain-dependent tolerance *r*. The whole process is repeated for (*m*+1)-length segments. Then, it is computed the conditional probability that two segments that are similar for *m* contiguous points remain similar when the run length increases to *m*+1. Finally, in order to provide a non-negative measure of entropy, SampEn is computed as the negative logarithm of such conditional probability using equation 1, so that higher values of SampEn are indicative of less similarity among patterns within the time series, i.e., higher irregularity or randomness [38].

SampEn is largely independent of the recording length and has been found to show relative large consistency in different frameworks [16,21,38]. Nevertheless, when computing SampEn, it is essential to properly set the input parameters *m*, i.e., the length of the segments to be compared, and *r*, i.e., the tolerance width for assessing similarity. In the present research, *m* = 1, 2, and 3 were assessed, which are commonly used values for HRV analysis [18,21]. Regarding the tolerance, it is convenient to modulate its width as *r* times the standard deviation (SD) of the time series in order to capture the characteristics of each particular application [38,39]. In the present study, *r* = 0.10, 0.15, 0.20, and 0.25 times the SD of the PRV signal were assessed, as this are the values originally proposed to obtain reproducible and consistent entropy measures [39].

### 2.3. Statistical Analysis

For each variable involved in the study, a descriptive analysis was carried out in terms of median and interquartile range (IQR). Additionally, the non-parametric Kruskall-Wallis test was used to search for statistical differences among the groups under study. The Fisher’s Least Significant Difference procedure was applied to correct for multiple comparisons. All *p*-values <0.05 were considered statistically significant. Linear association was assessed by means of the non-parametric Spearman correlation test. Statistical analyses were performed using IMB SPSS Statistics 20.0 (IBM Corp, Armonk, NY, USA) and MATLAB R2015a (The MathWorks Inc., Natick, MA, USA).

## 3. Results

Table 3 summarises the results from conventional time and spectral analyses of overnight PRV time series. Regarding variables in the time domain, patients with COPD and moderate-to-severe OSAS showed significantly lower AVNN or pulse-to-pulse interval (higher pulse rate) during the night than OSAS patients without COPD. On the other hand, COPD patients with and without moderate-to-severe OSAS did not show significant differences in terms of AVNN. Similarly, no significant differences between groups were found for SDNN and RMSSD, suggesting that the presence of OSAS among COPD patients and the presence of COPD among OSAS patients does not influence globally PRV during the night.

**Figure 1:**
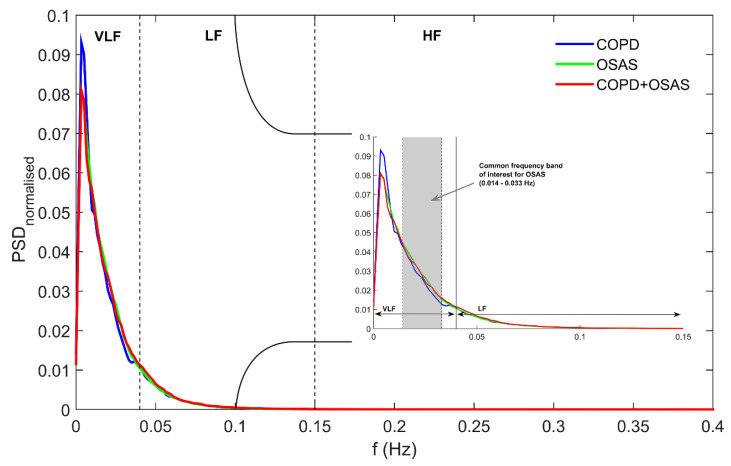
Normalised power spectrum for COPD (blue), OSAS (green), and COPD+OSAS (red) groups. For each group, the PSD is obtained as the average across all the patients.

Figure 1 shows the normalised power spectra averaged for all the patients within each group. We can observe that PSDs of overnight PRV recording from both OSAS-related groups (OSAS and COPD+OSAS patients) showed similar power spectral distribution while COPD patients without OSAS showed a different profile, particularly in the VLF band. The common OSAS-related frequency band of interest (0.014–0.033 Hz) is highlighted (light grey), where we found the common higher spectral power in both the OSAS and the COPD + OSAS groups linked with the recurrent apnoeic episodes along the night. Regarding variables from conventional frequency bands in Table 3, OSAS patients showed higher VLFn, LFn, and LF/HF, as well lower HFn than COPD patients without the disease, although no significant differences were found. Similarly, OSAS patients had significantly higher (*p* < 0.05) LFn, and LF/HF, as well lower HFn, than COPD + OSAS patients. Conventional time- and frequency- domain measures yielded no significant differences between overnight PRV recordings from COPD and COPD + OSAS patients. On the contrary, focusing on the OSAS-related frequency band of interest, COPD patients without OSAS showed significantly lower (*p* < 0.05) spectral power (lower OSASFn) than COPD + OSAS patients. Similarly, the COPD group also showed significantly lower OSASFn than the OSAS group.

**Figure 2:**
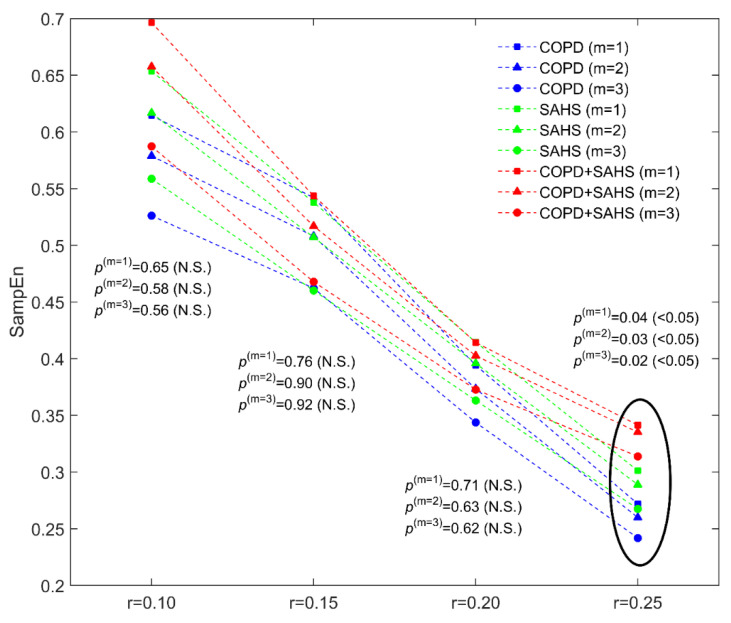
Changes in SampEn varying the domain-dependent input parameters *m* and *r* for the groups under study. All *p*-values are obtained after correction for multiple comparisons.

**Figure 3:**
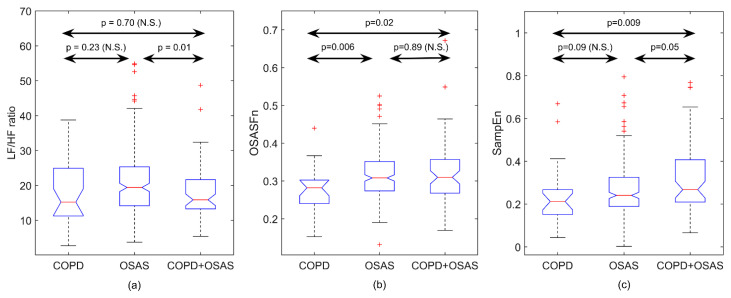
Boxplots showing the distribution of (**a**) LF/HF ratio, (**b**) spectral power in the OSAS-related frequency band, and (**c**) SampEn for the groups under study. All *p*-values are obtained after correction for multiple comparisons.

Regarding non-linear analysis, Figure 2 shows the influence of changes in input parameters *m* and *r* on the computation of SampEn for the quantification of irregularity of overnight PRV recordings. Lower values of both the pattern length and the tolerance were not able to capture differences among the groups under study. On the other hand, higher values of *m* and *r* maximises the differences between each pair of patient groups. Particularly, the highest statistical significant differences were reached for *m* = 3 and *r* = 0.25 times SD. Using this parameter setting, COPD patients with OSAS showed significantly higher SampEn than COPD patients without the disease. Furthermore, patients with both COPD and OSAS showed significantly increased SampEn than patients with OSAS alone. It is important to highlight a trend toward higher entropy values, i.e., higher irregularity, firstly from COPD to OSAS alone and, secondly, from OSAS to COPD+OSAS (overlap syndrome). Figure 3 illustrates this trend for SampEn, which is also observed for OSASFn. On the contrary, when using common spectral measures in the traditional frequency bands, patients with both COPD and OSAS showed slightly higher (non-significant) values than COPD patients but significantly lower than subjects suffering from OSAS alone.

No index from cardiac autonomic function assessment was significantly correlated with FEV_1_. Regarding correlation with OSAS severity, SDNN (*Rho* = 0.215; *p* < 0.01), VLFn (*Rho* = 0.148; *p* < 0.05), LFn (*Rho* = 0.161; *p* < 0.01), HFn (*Rho* = 0.161; *p* < 0.01), LF/HF (*Rho* = 0.161; *p* < 0.01), and OSASFn (*Rho* = 0.263; *p* < 0.01) were all significantly correlated with AHI. Similarly, there is a small but significant correlation with the ODI3 for AVNN (*Rho* = −0.143; *p* < 0.05), SDNN (*Rho* = 0.202; *p* < 0.01), VLFn (*Rho* = 0.126; *p* < 0.05), LFn (*Rho* = 0.161; *p* < 0.01), HFn (*Rho* = 0.161; *p* < 0.01), LF/HF (*Rho* = 0.161; *p* < 0.01), and OSASFn (*Rho* = 0.270; *p* < 0.01). On the other hand, no significant correlation was found between SampEn and FEV_1_ (*Rho* = -0.054; *p* = 0.627), AHI (*Rho* = 0.067; *p* = 0.251), or ODI3 (*Rho* = 0.050; *p* = 0.387).

## 4. Discussion

In this study, time, frequency and nonlinear measures are used to characterize the influence of COPD and OSAS on overnight cardiac modulation, both separately and together (overlap syndrome). Conventional variables in the time domain were computed to measure overall changes in PRV, while the traditional frequency bands (VLF, LF and HF) were used to assess changes in sympathetic and parasympathetic activity during the night linked with the presence of these diseases. On the other hand, the frequency band directly related to apnoeic pseudo-periodicity, as well as the long-term irregularity (SampEn) of the overnight PRV profile, were analysed to gain insight into the mechanisms elicited by both diseases.

Our analyses revealed that overnight PRV recordings from COPD + OSAS patients were significantly more irregular (higher SampEn) than those from patients with COPD alone (0.267 vs. 0.212; *p* < 0.05). Similarly, COPD + OSAS patients also showed significantly higher irregularity in PRV during the night than subjects with OSAS alone (0.267 vs. 0.241; *p* =0.05). In addition, nocturnal PRV recordings from OSAS patients both with and without COPD showed significantly higher spectral power in the OSAS-related frequency band than patients with COPD alone (0.310 vs. 0.282 and 0.308 vs. 0.282; *p* < 0.05). It is noticeable that spectral features from the conventional frequency bands VLF, LF, and HF were not able to identify differences between COPD patients with and without OSAS.

Regarding correlation analysis, our results suggest that increasing airflow limitation do not proportionally diminish cardiac autonomic function. On the contrary, the number of apnoeic/desaturation events per hour of sleep seems to influence conventional time- and frequency-domain measures of nocturnal cardiac modulation. However, increasing AHI of moderate-to-severe OSAS patients do not lead to increasing long-term disorderliness or irregularity, i.e., entropy. It is noticeable that SampEn was significantly correlated with OSASFn (*Rho* = 0.135; *p* < 0.05), which agrees with the trend to higher values of both indices due to the combined presence of COPD and moderate-to-severe OSAS regardless the particular severity of each condition. Therefore, both OSASFn and SampEn seems to better characterise the combined influence of COPD and OSAS on overnight PRV.

There is an increasing demand for controlling the influence of potentially relevant conditions in COPD patients, especially regarding cardiovascular performance [13]. In this way, some studies reported significant changes in cardiovascular dynamics of COPD patients during exercise and while sleeping, as well as during common acute exacerbations and in response to medication [11,40]. As OSAS is a frequent comorbid condition among COPD patients, recent reports strongly recommend screening for sleep related breathing disorders in COPD patients showing common symptoms [4,7,8]. The recurrent progressive-bradycardia/abrupt-tachycardia pattern during apnoeic episodes typical of OSAS have major haemodynamic and cardiovascular consequences [41,42], both during wakefulness and sleep [43].

It is well-known that OSAS is linked with increased sympathetic activation of the autonomous nervous system [7]. OSAS patients show a marked shift of the sympathovagal balance towards higher sympathetic activity and diminished parasympathetic control [44]. In the same way, COPD has been related to diminished cardiac autonomic modulation and increased risk of developing cardiovascular diseases [45]. Particularly, COPD alone is linked with augmented sympathetic autonomic discharges due to sustained hypoxaemia [46]. Accordingly, significantly higher sympathetic activity (LFn) and sympathovagal balance (LF/HF) could be expected in overlap patients due to the cumulative effect of both diseases. However, there are limited evidences on this relationship [7]. In this regard, a recent study by Taranto-Montemurro et al. [10] reported that patients with overlap syndrome have higher daytime sympathetic activation of the nervous system compared with those with OSAS or COPD alone. Similarly, Lopes-Zangrando et al. [9] found that patients with both COPD and OSAS showed marked sympathetic modulation during wakefulness than subjects with COPD alone. In the present study, there were no significant differences between COPD and COPD+OSAS groups regarding neither sympathetic activity (LFn) nor parasympathetic activity (HFn) and sympathovagal balance (LF/FH). Moreover, in our sample, overlap patients showed significantly lower sympathetic activity and sympathovagal balance as well as significantly higher parasympathetic activity than patients with OSAS alone. It is important to highlight at this point that all long-term overnight PRV recordings analysed in the present study were obtained during sleep. It is recognised that the presence of repetitive sleep apnoea episodes introduces a pseudo-periodic “biological noise” able to alter the autonomic cardiovascular modulations [43]. Recent studies reported increased vagal activation during NREM sleep [47] and decreased sympathetic modulation during REM sleep [48] in patients with higher AHI. Particularly, severe OSA has demonstrated to affect greatly HRV rhythmical oscillations introducing non-neural fluctuations that should be taken into account when interpreting the cardiovascular autonomic assessment during sleep [49]. When analysing nocturnal PRV recordings containing apnoeic events, the common power increase in the OSAS-related frequency band appears both in OSAS and in COPD + OSAS patients. Nevertheless, our results suggest a trend towards a higher impact of these events in overlap patients, which leads to a shift towards “non-OSAS conditions” in the remaining traditional spectral bands, thus covering the actual higher impact of apnoea events in the presence of COPD. These hidden spectral differences between COPD and COPD+OSAS patients arise when resting short-term (10 min) daytime ECGs are analysed during wakefulness without the direct interference of apnoeas [9,10]. Similarly, Kabbach et al. [12] found notably higher parasympathetic cardiac autonomic modulation in COPD exacerbated patients. Under these particular conditions, they reported that higher HF power was linked with airway narrowing during exacerbations and should not be interpreted as a better clinical condition. In this sense, the LFn decrease and HFn increase we found in nocturnal PRV recordings from COPD + OSAS patients compared to OSAS alone should not be seen as an improvement in the patient’s health status but as a worsening due to recurrent airway obstruction during the night.

On the other hand, long-term non-linear analysis of whole-night PRV recordings matches the results obtained during wakefulness, reinforcing the idea of diminished cardiovascular balance (higher dysfunction) due to the cumulative effect of both diseases. It is commonly assumed that entropy of heart rate is lower in patients with cardiovascular diseases than in healthy people [11], i.e., lower entropy or irregularity is a marker of diseased states. In this regard, previous studies in the context of COPD reported decreased entropy of HRV during respiratory sinus arrhythmia [23,50] and during exacerbations [12]. Similarly, Lopes-Zangrando et al. [9] reported lower non-linear indices derived from HRV recordings in COPD+OSAS patients compared with patients with COPD alone. However, it is important to highlight that this assumption depends on the condition under study (disease mechanisms, wakefulness/sleep) and on the characteristics of the physiological signal (specially the length of the time series) [11]. Table 4 summarises the main characteristics of the studies from the state-of-the-art using non-linear analysis to assess HRV in the framework of COPD. Iranmanesh et al. [51] reported that hormone secretion showed higher irregularity (higher entropy) in COPD patients. Similarly, the entropy of respiratory sounds has been found to be higher in non-healthy subjects [36] and the entropy of mechanomyogram signals is also higher as the severity of COPD increases [52]. In the presence of OSAS, different studies reported higher non-linear measures values of overnight oximetry [33,34], airflow [35], and heart rate [21,26,53] derived from whole-night PSG. In these studies, it has been found that direct presence of apnoeic events modifies the normal cardiorespiratory dynamics towards higher disorderliness or disorganization, i.e., higher entropy. Consequently, in the present study, significantly higher SampEn has been found in patients with concomitant COPD and OSAS compared to patients with COPD alone. Furthermore, overlap patients also showed significantly higher SampEn than patients with OSAS alone. This agrees with a previous study by Zamarrón et al. [26], who reported significantly higher entropy values in patients with both OSAS and COPD compared to OSAS patients without COPD. Nevertheless, no extensive comparison with a group of patients with COPD alone was carried out.

Several limitations should be taken into account. Firstly, although our population under study is notably large compared to previous similar studies, a larger dataset would increase the significance and generalizability of our findings. Additionally, there is an imbalance in the number of individuals within the groups under study, which could influence the results. Particularly, a higher number of COPD patients without moderate-to-severe OSAS would be needed to enhance the characterisation of such patient group. In this regard, it is important to highlight that all patients showed moderate-to-high clinical suspicion of sleep disordered breathing. As OSAS is frequent among COPD patients, the probability of suffering from COPD alone was low in our sample. Another limitation arises concerning the groups under study. A control group composed of healthy patients was not analysed in the present study due to the fact all participants reported daily and/or nocturnal symptoms of sleep apnoea. Although this study focuses on HRV analysis in the presence of disease, particularly the cumulative effect of COPD and OSAS, the inclusion of healthy individuals would be interesting to set reference values of HRV dynamics for all the proposed signal processing approaches. An additional drawback arises regarding the potential presence of COPD among patients within the moderate-to-severe OSAS group. According to our inclusion criteria, these patients showed no history of COPD in their clinical records and had no respiratory symptoms indicative of suffering from the disease. However, a post-bronchodilator spirometry would be needed to completely discard COPD. Regarding demographic characteristics, male gender was significantly higher in all the patient groups. In addition, significant differences between healthy men and women has been found in HRV [54] suggesting that an exhaustive analysis of gender-related specificities would be needed to assess such potential differences also under diseased conditions, such as COPD. Moreover, patients in the COPD + OSAS group were older than patients with COPD or OSAS alone. However, a consistent entropy (complexity/irregularity) loss has been reported with aging [19], while we observed that patients with overlap syndrome reached significantly higher SampEn than younger individuals with OSAS or COPD alone. Therefore, it is unlikely that differences in entropy of overnight PRV recordings were driven by increased age. On average, COPD patients, both with and without OSAS, showed basal SpO_2_ values slightly higher than 90% (92% and 91%, respectively), which is a common threshold for considering hypoxaemia. Therefore, it would be interesting to include COPD patients showing higher levels of hypoxaemia in future works in order to generalise our results. Finally, the potential influence of medication must be mentioned. COPD and overlap patients frequently use anticholinergics and β2-agonists. Currently, there is controversy on the effect of this medication on the autonomic balance [55,56], so extensive research is still needed in order to assess its influence on HRV dynamics, particularly in the presence of OSAS.

## 5. Conclusions

Non-linear analysis of nocturnal PRV recordings has been found to provide further insight into the differences between COPD patients without and with OSAS (overlap syndrome). Conventional spectral measures in the traditional frequency bands LF and HF were unable to capture changes in overnight HRV dynamics linked with apnoeic events in COPD patients while sleeping. On the contrary, we found that patients with both COPD and OSAS showed significantly increased SampEn (higher irregularity) than patients showing COPD or OSAS alone. Therefore, our findings suggest that there is a cumulative effect of COPD and OSAS towards increased cardiovascular dysfunction. We conclude that SampEn is able to properly quantify changes in overnight PRV dynamics of patients with overlap syndrome, which could be useful to assess cardiovascular impairment in COPD patients due to the presence of concomitant OSAS.

## Figures and Tables

**Table 1:** Demographic, polysomnographic, and spirometric variables of the groups under study.

Demographics	COPD	OSAS	COPD + OSAS	*p*-Value
Nº of subjects	22	213	62	-
Nº of males (%)	16 (72.7%)	167 (78.4%)	58 (93.6%)	-
Age (years)	60.5 (57, 64) ^¶^	56 (47, 64) ^†^	66 (60, 75) ^¶†^	<0.05
BMI (kg/m^2^)	27.3 (24.3, 29.4)	29.4 (26.7, 32.8)	29.6 (26.8, 32.7)	N.S.
**Polysomnography**				
AHI (events/h)	9.0 (5.2, 11.1) ^*¶^	44.2 (30.6, 66.5) ^*^	53.7 (29.0, 67.2) ^¶^	<0.05
ODI3 (events/h)	9.9 (5.3, 11.4) ^*¶^	42.7 (30.6, 62.4) ^*^	51.6 (31.7, 64.4) ^¶^	<0.05
CT90 (%)	22.9 (0.4, 87.3) ^¶^	14.3 (6.3, 39.7) ^†^	47.8 (24.7, 88.2) ^¶†^	<0.05
SpO2_basal_ (%)	92 (90, 94) ^*^	94 (93, 94) ^*†^	91 (90, 93) ^†^	<0.05
SpO2_min_ (%)	85 (83, 87) ^*¶^	80 (73, 83) ^*†^	76.5 (71, 81) ^¶†^	<0.05
SpO2_avg_ (%)	91.5 (89, 94) ^¶^	92 (90, 93) ^†^	90 (88, 92) ^¶†^	<0.05
**Pulmonary Function (Spirometry)**				
FVC (liters)	3.2 (2.3, 3.8)	N.A.	2.6 (2.2, 3.5)	N.S.
FVC (%)	91 (72.8, 100)	N.A.	77.3 (67, 92)	<0.05
FEV_1_ (liters)	1.9 (1.5, 2.2)	N.A.	1.6 (1.3, 2.1)	N.S.
FEV_1_ (%)	68.5 (55.0, 83.2)	N.A.	62.5 (54.3, 73.0)	N.S.
FEV_1_/FVC	59.4 (50.1, 65.2)	N.A.	60.9 (52.0, 65.4)	N.S.
FVC improvement	3.3 (2.6, 11.0)	N.A.	3.9 (2.5, 7.3)	N.S.
FEV_1_ improvement	3.9 (2, 8)	N.A.	3 (1.6, 5.3)	N.S.

BMI: body mass index; COPD: chronic obstructive pulmonary disease; CT90: cumulative time with a saturation below 90%; FEV_1_: forced expired volume in 1 s; FVC: forced vital capacity; ODI3: oxygen desaturation index of 3%; OSAS: obstructive sleep apnoea syndrome; SpO2_avg_: average overnight blood oxygen saturation; SpO2_basal_: baseline blood oxygen saturation; SpO2_min_: minimum overnight blood oxygen saturation. Data is provided as median (interquartile range) or n (%). ^*^ Significant differences between COPD and OSAS groups (*p* < 0.05). ^¶^ Significant differences between COPD and COPD + OSAS groups (*p* < 0.05). ^†^ Significant differences between OSAS and COPD + OSAS groups (*p* < 0.05).

**Table 2:** Comorbidities and therapies in the study population.

	COPD (22)	OSAS (213)	COPD+OSAS (62)	*p*-Value
**Comorbidities**				
Hypertension, n (%)	10 (45.5%)	88 (41.3%) ^†^	42 (67.7%) ^†^	<0.05
Ischemic Cardiomyopathy, n (%)	2 (9.1%)	11 (5.2%) ^†^	10 (16.1%) ^†^	<0.05
Diabetes, n (%)	3 (13.6%)	22 (10.3%)	9 (14.5%)	N.S.
**Medications**				
Beta-blockers, n (%)	2 (9.1%)	25 (11.7%) ^†^	15 (24.2%) ^†^	<0.05
Calcium antagonists, n (%)	1 (4.5%)	18 (8.5%)	6 (9.7%)	N.S.
Anticholinergics, n (%)	8 (36.4%)	1 (0.5%)	36 (58.1%)	N.S. ^ǂ^
Beta2-Agonists, n (%)	8 (36.4%)	8 (3.8%)	29 (46.8%)	N.S. ^ǂ^
Inhaled corticosteroids, n (%)	7 (31.8%)	9 (4.2%)	27 (43.6%)	N.S. ^ǂ^

^*^ Significant differences between COPD and OSAS groups (*p* < 0.05). ^¶^ Significant differences between COPD and COPD+OSAS groups (*p* < 0.05). ^†^ Significant differences between OSAS and COPD + OSAS groups (*p* < 0.05). ^ǂ^ Comparison only between COPD and COPD + OSAS groups.

**Table 3:** Time, frequency and nonlinear PRV measures for the patient groups under study.

	COPD	OSAS	COPD + OSAS	*p*-Value
**Time domain analysis**				
AVNN	0.873 (0.825, 0.980)	0.961 (0.852, 1.051) ^†^	0.906 (0.787, 1.022) ^†^	0.026 (*p* < 0.05)
SDNN	0.066 (0.058, 0.074)	0.075 (0.062, 0.094)	0.071 (0.056, 0.090)	0.088 (N.S.)
RMSSD (10^−4^)	0.577 (0.466, 0.756)	0.724 (0.578, 0.863)	0.686 (0.522, 0.831)	0.071 (N.S.)
**Frequency Domain Analysis**				
VLFn	0.802 (0.774, 0.825)	0.821 (0.792, 0.844)	0.812 (0.772, 0.844)	0.061 (N.S.)
LFn	0.938 (0.931, 0.961)	0.951 (0.934, 0.962) ^†^	0.941 (0.930, 0.956) ^†^	0.028 (*p* < 0.05)
HFn	0.062 (0.039, 0.069)	0.049 (0.038, 0.066) ^†^	0.059 (0.044, 0.070) ^†^	0.028 (*p* < 0.05)
LF/HF	15.243 (13.569, 24.926)	19.419 (14.179, 25.343) ^†^	15.881 (13.301, 21.673) ^†^	0.028 (*p* < 0.05)
OSASFn	0.282 (0.240, 0.302) ^*¶^	0.308 (0.274, 0.351) ^*^	0.310 (0.268, 0.357) ^¶^	0.022 (*p* < 0.05)
**Nonlinear Analysis**				
SampEn	0.212 (0.151, 0.267) ^¶^	0.241 (0.189, 0.325) ^†^	0.267 (0.210, 0.407) ^¶†^	0.022 (*p* < 0.05)

AVNN: Average of pulse-to-pulse interval; COPD: chronic obstructive pulmonary disease; HFn: normalised spectral power in the high frequency band; LF/HF: low frequency to high frequency ratio or sympathovagal balance; LFn: normalised spectral power in the low frequency band; OSAS: obstructive sleep apnoea syndrome; OSASFn: normalised spectral power in the apnoea-related frequency band; RMSSD: Root mean square of successive differences of pulse-to-pulse intervals; SampEn: sample entropy; SDNN: standard deviation of pulse-to-pulse interval; VLFn: normalised spectral power in the very low frequency band. Data is provided as median (interquartile range). ^*^ Significant differences between COPD and OSAS groups (*p* < 0.05). ^¶^ Significant differences between COPD and COPD + OSAS groups (*p* < 0.05). ^†^ Significant differences between OSAS and COPD+OSAS groups (*p* < 0.05).

**Table 4:** Summary of the state-of-the-art of studies focused on non-linear analysis of HRV recordings in the framework of COPD.

Author	Population	Aim	HRV Acquisition Setting	Non-Linear Analysis	Findings
Zamarrón et al. (2006) [26]	187 suspicion OSAS: - 76 no-OSAS - 89 OSAS - 22 overlap	PRV assessment in OSAS patients	Night-time while sleeping (attended)	ApEn	Increased irregularity (ApEn) in overlap patients
Borghi-Silva et al. (2015) [24]	20 COPD	Effect of physical training on HRV in COPD	Daytime at rest	SampEn	HRV irregularity increases after physical training
Mazzuco et al. (2015) [50]	16 COPD	HRV assessment in increasing COPD severity	Daytime at rest	ApEn	HRV irregularity decreases during RSA
Goulart et al. (2016) [23]	10 COPD	HRV assessment in COPD	Daytime at rest	SampEn and ApEn	HRV irregularity decreases during RSA
Kabbach et al. (2017) [12]	32 COPD: - 16 stable - 16 exacerbated	HRV assessment in COPD with and without exacerbation	Daytime at rest	ApEn, SampEn, and scatter plots	Irregularity (entropy) decreases while variability (dispersion) increases after exacerbation
Zangrando et al. (2018) [9]	24 COPD: - 12 COPD - 12 overlap	HRV assessment in COPD	Daytime at rest	Scatter plots	Higher (N.S.) variability in overlap patients
Current study (2019)	297 suspicion OSAS: - 22 COPD - 213 OSAS - 62 overlap	PRV assessment in COPD patients with and without OSAS	Night-time while sleeping (unattended)	SampEn	Increased irregularity (SampEn) in overlap patients

ApEn: approximate entropy; COPD: chronic obstructive pulmonary disease; HRV: heart rate variability; OSAS: obstructive sleep apnoea; PRV: pulse rate variability; RSA: respiratory sinus arrhythmia; SampEn: sample entropy.

## References

[1] Vogelmeier C.F., Criner G.J., Martinez F.J., Anzueto A., Barnes P.J., Bourbeau J., Celli B.R., Chen R., Decramer M. (2017). Global Strategy for the Diagnosis, Management and Prevention of Chronic Obstructive Lung Disease 2017 Report. Respirology.

[2] Halbert R.J., Natoli J.L., Gano A., Badamgarav E., Buist A.S., Mannino D.M. (2006). Global burden of COPD: systematic review and meta-analysis. Eur. Resp. J..

[3] Chronic Respiratory Disease Collaborators (2017). Global, regional, and national deaths, prevalence, disability-adjusted life years, and years lived with disability for chronic obstructive pulmonary disease and asthma, 1990– 2015: A systematic analysis for the Global Burden of Disease Study (GBD 2015). Lancet Respir. Med..

[4] Hang L.-W., Hsu J.-Y., Chang C.-J., Wang H.-C., Cheng S.-L., Lin C.-H., Chan M.-C., Wang C.-C., Perng D.-W., Yu C.-J. (2016). Predictive factors warrant screening for obstructive sleep apnea in COPD: A Taiwan National Survey. Int. J. COPD.

[5] Müllerova H., Agustí A., Erqou S., Mapel D.W. (2013). Cardiovascular Comorbidity in COPD: Systematic Literature Review. Chest.

[6] McNicholas W.T. (2009). Chronic Obstructive Pulmonary Disease and Obstructive Sleep Apnea: Overlaps in Pathophysiology, Systemic Inflammation, and Cardiovascular Disease. Am. J. Respir. Crit. Care Med..

[7] McNicholas W.T. (2017). COPD-OSA Overlap Syndrome: evolving evidence regarding epidemiology, clinical consequences, and management. Chest.

[8] Mieczkowski B., Ezzie M.E. (2014). Update on obstructive sleep apnea and its relation to COPD. Int. J. COPD.

[9] Lopes Zangrando K.T., Trimer R., Soares de Carvalho L.C., Tinoco Arêas G.P., Rossi Caruso F.C., Cabiddu R., Goi Roscani M., Galhardo Rizzatti F.P., Borghi-Silva A. (2018). Chronic obstructive pulmonary disease severity and its association with obstructive sleep apnea syndrome: impact on cardiac autonomic modulation and functional capacity. Int. J. COPD.

[10] Taranto-Montemurro L., Messineo L., Perger E., Salameh M., Pini L., Corda L., Ferliga M., Tantucci C. (2016). Cardiac Sympathetic Hyperactivity in Patients with Chronic Obstructive Pulmonary Disease and Obstructive Sleep Apnea. COPD J. Chronic Obstr. Pulm. Dis..

[11] Jin Y., Chen C., Cao Z., Sun B., Lo I.L., Liu T.-M., Zheng J., Sun S., Shi Y., Zhang X.D. (2017). Entropy change of biological dynamics in COPD. Int. J. COPD.

[12] Kabbach E.Z., Mazzuco A., Borghi-Silva A., Cabiddu R., Agnoleto A.G., Barbosa J.F., Soares de Carvalho L.C., Gonçalves Mendes R. (2017). Increased parasympathetic cardiac modulation in patients with acute exacerbation of COPD: how should we interpret it?. Int. J. COPD.

[13] Malhotra A., Schwartz A.R., Schneider H., Owens R.L., De Young P., Han M.K., Wedzicha J.A., Hansel N.N., Zeidler M.R., Wilson K.C. (2018). On behalf of the ATS Assembly on Sleep and Respiratory Neurobiology. Research Priorities in Pathophysiology for Sleep-disordered Breathing in Patients with Chronic Obstructive Pulmonary Disease An Official American Thoracic Society Research Statement. Am. J. Respir. Crit. Care Med..

[14] Thayer J.F., Yamamoto S.S., Brosschot J.F. (2010). The relationship of autonomic imbalance, heart rate variability and cardiovascular disease risk factors. Int. J. Cardiol..

[15] Stein P.K., Pu Y. (2012). Heart rate variability, sleep and sleep disorders. Sleep Med. Rev..

[16] Abásolo D., Hornero R., Espino P., Álvarez D., Poza J. (2006). Entropy analysis of the EEG background activity in Alzheimer’s disease patients. Physiol. Meas..

[17] Chang Y.C., Wu H.T., Chen H.R., Liu A.B., Yeh J.J., Lo M.T., Tsao J.H., Tang C.-J., Tsai I.-T., Sun C.-K. (2014). Application of a Modified Entropy Computational Method in Assessing the Complexity of Pulse Wave Velocity Signals in Healthy and Diabetic Subjects. Entropy.

[18] Alcaraz R., Rieta J.J. (2009). Sample entropy of the main atrial wave predicts spontaneous termination of paroxysmal atrial fibrillation. Med. Eng. Phys..

[19] Costa M.D., Goldberger A.L., Peng C.K. (2005). Multiscale entropy analysis of biological signals. Phys. Rev. E.

[20] Álvarez D., Hornero R., Marcos J.V., Del Campo F. (2010). Multivariate analysis of blood oxygen saturation recordings in obstructive sleep apnea diagnosis. IEEE Trans. Biomed. Eng..

[21] Gutiérrez-Tobal G.C., Álvarez D., Gomez-Pilar J., Del Campo F., Hornero R. (2015). Assessment of time and frequency domain entropies to detect sleep apnoea in heart rate variability recordings from men and women. Entropy.

[22] Al-Angari H.M., Sahakian A.V. (2007). Use of sample entropy approach to study heart rate variability in obstructive sleep apnea syndrome. IEEE Trans. Biomed. Eng..

[23] Goulart C.L., Simon J.C., Schneiders P.B., San Martin E.A., Borghi-Silva A., Trimer R., de Silva A.L. (2016). Respiratory muscle strength effect on linear and nonlinear heart rate variability parameters in COPD patients. Int. J. COPD.

[24] Borghi-Silva A., Mendes R.G., Trimer R., Oliveira C.R., Fregonezi G.A., Resqueti V.R., Arena R., Sampaio-Jorge L.M., Costa D. (2015). Potential effect of 6 versus 12-weeks of physical training on cardiac autonomic function and exercise capacity in chronic obstructive pulmonary disease. Eur. J. Phys. Rehabil. Med..

[25] Dames K.K., Lopes A.J., De Melo P.L. (2014). Airflow pattern complexity during resting breathing in patients with COPD: effect of airway obstruction. Respir. Physiol. Neurobiol..

[26] Zamarrón C., Hornero R., Del Campo F., Abásolo D., Alvarez D. (2006). Heart rate regularity analysis obtained from pulse oximetric recordings in the diagnosis of obstructive sleep apnea. Sleep Breath..

[27] Iber C., Ancoli-Israel S., Chesson A., Quan S.F. (2007). For the American Academy of Sleep Medicine. The AASM Manual for the Scoring of Sleep and Associated Events-Rules, Terminology and Technical Specifications.

[28] Zamarrón C., Gude F., Barcala J., Rodríguez J.R., Romero P.V. (2003). Utility of oxygen saturation and heart rate spectral analysis obtained from pulse oximetric recordings in the diagnosis of sleep apnea syndrome. Chest.

[29] Redline S., Sanders M.H., Lind B.K., Quan S.F., Iber C., Gottlieb D.J., Bonekat W.H., Rapoport D.M., Smith P.L., Kiley J.P. (1998). For the Sleep Heart Health Research Group. Methods for obtaining and analyzing unattended polysomnography data for a multicenter study. Sleep.

[30] Penzel T., Kantelhardt J.W., Grote L., Peter J.H., Bunde A. (2003). Comparison of detrended fluctuation analysis and spectral analysis for heart rate variability in sleep and sleep apnea. IEEE Trans. Biomed. Eng..

[31] Goldberger A.L. (1991). Is the normal heartbeat chaotic or homeostatic?. News Physiol. Sci..

[32] Wessel N., Riedl M., Kurths J. (2009). Is the normal heart rate “chaotic” due to respiration?. Chaos.

[33] Hornero R., Álvarez D., Abásolo D., Del Campo F., Zamarrón C. (2007). Utility of approximate entropy from overnight pulse oximetry data in the diagnosis of the obstructive sleep apnea syndrome. IEEE Trans. Biomed. Eng..

[34] Del Campo F., Hornero R., Zamarrón C., Abasolo D.E., Álvarez D. (2006). Oxygen saturation regularity analysis in the diagnosis of obstructive sleep apnea. Artif. Intell. Med..

[35] Gutiérrez-Tobal G.C., Álvarez D., Del Campo F., Hornero R. (2016). Utility of AdaBoost to detect sleep apnea-hypopnea syndrome from single-channel airflow. IEEE Trans. Biomed. Eng..

[36] Mondal A., Bhattacharyat P., Saha G. Diagnosing of the lungs status using morphological anomalies of the signals in transformed domain. Proceedings of the 4th International Conference on Intelligent Human Computer Interaction.

[37] Garde A., Dehkordi P., Karlen W., Wensley D., Ansermino J.M., Dumont G.A. (2014). Development of a screening tool for sleep disordered breathing in children using the Phone OximeterTM. PLoS ONE.

[38] Richman J.S., Moorman J.R. (2000). Physiological time series analysis using approximate entropy and sample entropy. Am. J. Physiol. Heart Circ. Physiol..

[39] Pincus S.M., Goldberger A.L. (1994). Physiological time series analysis: what does regularity quantify?. Am. J. Physiol..

[40] Zamarrón C., Lado M.J., Teijeiro T., Morete E., Vila X.A., Lamas P.F. (2014). Heart rate variability in patients with severe chronic obstructive pulmonary disease in a home care program. Technol. Health Care.

[41] Guilleminault C., Winkle R., Connolly S., Melvin K., Tilkian A. (1984). Cyclical variation of the heart rate in sleep apnoea syndrome: Mechanisms and usefulness of 24 h electrocardiography as a screening technique. Lancet.

[42] Bonsignore M.R., Romano S., Marrone O., Chiodi M., Bonsignore G. (1997). Different heart rate patterns in obstructive apneas during NREM sleep. Sleep.

[43] Tobaldini E., Nobili L., Strada S., Casali K.R., Braghiroli A., Montano N. (2013). Heart rate variability in normal and pathological sleep. Front. Physiol..

[44] Narkiewicz K., Montano N., Cogliati C., Van de Borne P.J.H., Dyken M.E.D., Somers V.K. (1998). Altered cardiovascular variability in obstructive sleep apnea. Circulation.

[45] Gestel A.J.R., Steier J. (2010). Autonomic dysfunction in patients with chronic obstructive pulmonary disease (COPD). J. Thorac. Dis..

[46] Heindl S., Lehnert M., Criee C.P., Hasenfuss G., Andreas S. (2001). Marked sympathetic activation in patients with chronic respiratory failure. Am. J. Resp. Crit. Care Med..

[47] Da Silva S.P., Hulce V.D., Backs R.W. (2009). Effects of obstructive sleep apnea on autonomic cardiac control during sleep. Sleep Breath..

[48] Gula L.J., Krahn A.D., Skanes A., Ferguson K.A., George C., Yee R. (2003). Heart rate variability in obstructive sleep apnea: a prospective study and frequency domain analysis. Ann. Noninvasive Electrocardiol..

[49] Wang W., Tretriluxana S., Redline S., Surovec S., Gottlieb D.J., Khoo M.C. (2008). Association of cardiac autonomic function measures with severity of sleep-disordered breathing in a community-based sample. J. Sleep Res..

[50] Mazzuco A., Medeiros W.M., Sperling M.P. (2015). Relationship between linear and nonlinear dynamics of heart rate and impairment of lung function in COPD patients. Int. J. COPD.

[51] Iranmanesh A., Rochester D.F., Liu J., Veldhuis J.D. (2011). Impaired adrenergic- and corticotropic-axis outflow during exercise in chronic obstructive pulmonary disease. Metabolism.

[52] Sarlabous L., Torres A., Fiz J.A., Jane R. (2014). Evidence towards improved estimation of respiratory muscle effort from diaphragm mechanomyographic signals with cardiac vibration interference using sample entropy with fixed tolerance values. PLoS ONE.

[53] Del Campo F., Hornero R., Zamarrón C., Álvarez D., Marcos J.V. (2010). Variability of pulse signal frequency obtained using nocturnal pulse oximetry in patients with sleep apnoea/hypoapnoea syndrome. Arch. Bronconeumol..

[54] Bonnemeier H., Wiegand U.K., Brandes A., Kluge N., Katus H.A., Richardt G., Potratz J. (2003). Circadian profile of cardiac autonomic nervous modulation in healthy subjects. J. Cardiovasc. Electrophysiol..

[55] Haarmann H., Mohrlang C., Tschiesner U., Rubin D.B., Bornemann T., Rüter K., Bonev S., Raupach T., Hasenfuß G., Andreas S. (2015). Inhaled β-agonist does not modify sympathetic activity in patients with COPD. BMC Pulm. Med..

[56] Wu Y.K., Huang C.Y., Yang M.C., Huang G.L., Chen S.Y., Lan C.C. (2015). Effect of tiotropium on heart rate variability in stable chronic obstructive pulmonary disease patients. J. Aerosol Med. Pulm. Drug Deliv..

